# Driven early detection of chronic kidney cancer disease based on machine learning technique

**DOI:** 10.1371/journal.pone.0326080

**Published:** 2025-07-15

**Authors:** Wafa Almukadi, Sayed Abdel-Khalek, Adel A. Bahaddad, Ahmed Mohammed Alghamdi

**Affiliations:** 1 Department of Software Engineering, College of Computer Science and Engineering, University of Jeddah, Jeddah, Saudi Arabia; 2 Mathematics Department, Faculty of Science, Sohag University, Sohag, Egypt; 3 Department of Information System, Faculty of Computing and Information Technology, King Abdulaziz University Jeddah, Jeddah, Saudi Arabia; South China University of Technology, CHINA

## Abstract

In recent times, chronic kidney cancer has been considered a significant cause of cancer, and Renal Cell Carcinoma (RCC) has become a significant prevalent among various kidney cancer conditions. The analysis of kidney cancer, an important and often time-sensitive medical task, has seen a breakthrough alteration by incorporating deep learning (DL) methods, mainly in analyzing histopathological images (HIs). Given manual analysis’s inherent complexity and time-consuming aspect, automatic systems leveraging DL methods provide a promising solution. Automated techniques powered by DL methods showcase a notable capability to analyze intricate details within HIs. These methods are adept at recognizing complex patterns and anomalies within HIs, accelerating the diagnostic method and increasing accuracy. The combination of advanced computational methods with the assessment of kidney cancer HIs not only overcomes the demanding requirements for timely identification but also paves the way for more effective and reliable diagnostic processes in renal oncology. This study presents the Kidney Cancer Detection and Classification employing a Snake Optimizer with Deep Learning on Pathological Images (KCDC-SODLPI) technique. The main aim of the KCDC-SODLPI method is to analyze the pathological images to determine the presence of kidney cancer. In the multifaceted process, the KCDC-SODLPI technique utilizes a Gaussian filtering (GF)-based image preprocessing approach to eliminate the noise content. Furthermore, the KCDC-SODLPI method employs the SE-DenseNet model for extracting intricate patterns from the input images. Moreover, the SO model is used to tune the hyperparameter of the SE-DenseNet method. Finally, the bidirectional long shortterm memory (BiLSTM) model is implemented to detect and classify kidney cancer. The performance of the KCDC-SODLPI technique is evaluated under the biomedical image dataset. The experimental validation of the KCDC-SODLPI method portrayed a superior accuracy value of 88.90% over existing models.

## 1. Introduction

Kidney cancer is a general kind of cancer, which is highly responsible for nearly 2.4 percent of cancer. The American Cancer Society calculates that 73,750 novel patients have been identified with this melanoma in the US, and around 14,830 persons will die of this illness [1]. RCC is a significantly prevalent kind and the reason for almost 85 percent of kidney cancer. RCC becomes an assorted type of cancer with dissimilar molecular features, reactions to treatment, histology, and medical results [2]. The most general sub-types of RCC are chromophobe (4–8%), clear cell (70–80%), papillary (14–17%), and clear cell papillary RCC (4%). These RCC sub-types can mainly depend upon the morphological feature experiential on histological training marked with eosin and hematoxylin (H&E strain) [3]. Compared to other sub-types, clear cell RCC and clear cell papillary RCC are the most critical morphologic overlay, particularly the common occurrence of clear cells. The difference between clear cell papillary RCC and clear cell RCC will be vital to define suitable patient administration. Clear cell RCC has a poor prediction owing to the higher danger of spreading with metastasis [4]. The physical ranking of complex histologic patterns of medical images is a tiresome challenge, and it is exposed to flaws and mistakes, so it needs an expert pathologist [5]. A complete automatic and exact model of ranking kidney tumours from HIs is a higher demand for classifying malignant cancers. DL is used to analyze the HIs of the liver, breast, kidney, and different parts, and it contains many tasks like nuclei segmentation, recognition and classification of cancer sub-types, and ranking. DL is the most significant machine learning (ML) technique, which can mechanically learn numerous patterns and features without human involvement [6].

DL enabled the construction of analytical methods for the initial analysis of cancer illness, and experts employed verified pattern analysis models. DL models have beaten classical ML owing to their higher accurate outcomes. Furthermore, it often equals or exceeds the performance of humans. So, they are suggested as the optimal model for imageries [7]. There has been an enormous awareness of image processing, particularly in the medical domain, because radiology was mainly affected by removing beneficial data from images. The convolutional neural network (CNN) was commonly employed to remove image features and identify dissimilar features. It functions as the source of weight sharing. The convolutional is a vital part that clarifies how a single function affects each other [8]. The dimension, amount of imageries, the number of functioning layers, and the method of activation function employed in CNN may differ. CNN variables are nominated experimentally on an experimental and error base. Also, each CNN contains numerous layers; the most significant are the pooling and convolution layers [9]. The complexity of RCC arises from its diverse subtypes, each with distinct molecular characteristics and treatment responses, making early detection and personalized treatment crucial. Accurate and timely detection of RCC is essential for improving patient outcomes, yet current diagnostic methods often rely on histological analysis, which can be subjective and time-consuming. The integration of ML techniques presents a promising solution for improving the precision and efficiency of early detection, enabling better prognosis and treatment planning for patients with kidney cancer [10].

This study presents the Kidney Cancer Detection and Classification employing a Snake Optimizer with Deep Learning on Pathological Images (KCDC-SODLPI) technique. The main aim of the KCDC-SODLPI method is to analyze the pathological images to determine the presence of kidney cancer. In the multifaceted process, the KCDC-SODLPI technique utilizes a Gaussian filtering (GF)-based image preprocessing approach to eliminate the noise content. Furthermore, the KCDC-SODLPI method employs the SE-DenseNet model for extracting intricate patterns from the input images. Moreover, the SO model is used to tune the hyperparameter of the SE-DenseNet method. Finally, the bidirectional long short-term memory (BiLSTM) model is implemented to detect and classify kidney cancer. The performance of the KCDC-SODLPI technique is evaluated under the biomedical image dataset. The major contribution of the KCDC-SODLPI technique is listed below.

- The KCDC-SODLPI model utilizes GF for image preprocessing, effectually mitigating noise and improving image quality. This improves the accuracy of kidney cancer detection in HIs. By ensuring more precise and reliable input, the model supports better analysis and classification of kidney cancer.
- The KCDC-SODLPI technique employs SE-DenseNet for feature extraction, allowing the model to capture intrinsic patterns from input images. This methodology improves the detection of subtle details that are significant for detecting kidney cancer. Utilizing this technique enhances the model’s ability to differentiate between normal and cancerous tissue in HIs.
- The KCDC-SODLPI methodology utilizes the SO method for hyperparameter tuning, improving the performance of the SE-DenseNet model. This optimization enhances the technique’s accuracy and efficiency in extracting relevant features from HIs. By fine-tuning the model, SO ensures more reliable and precise kidney cancer detection.
- The KCDC-SODLPI model utilizes the BiLSTM approach to detect and classify kidney cancer, improving its capability to capture both forward and backward temporal dependencies. This approach allows the model to better comprehend the sequential nature of histopathological patterns. As a result, the system becomes more accurate in detecting and classifying cancerous features in the images.
- The novelty of the KCDC-SODLPI technique is in its integration of advanced DL techniques, such as SE-DenseNet, BiLSTM, and SO. This fusion improves both the accuracy and effectiveness of analyzing HIs. By employing these cutting-edge approaches, the model efficiently addresses the complexity of cancer detection, providing a robust outcome for precise classification.

## 2. Related works

Mahootiha et al. [11] implemented a DL method comprising two networks such as a survival and classifier network. The 3D CNN method was employed. The technique also deployed the ISUP grading features extraction by the model as the input to the survival network. A DL-based method was also used. A discrete Logistic Hazard-based loss was also used to extract RCC cancer’s complex survival features from CT images. In [12], an innovative multiscale weakly-supervised DL method was employed. The technique was utilized to implement the RGB-histogram requirement stain normalization. Afterwards, the method follows the several-instance learning technique by separating the input data into numerous overlapping models for maintaining the tissue connections. In conclusion, three multiscale CNN methods have been trained, and the decision fusion method was implemented for the last classification. Hao et al. [13] implemented whole-slide images (WSIs) scanned through light microscopy and immune-fluorescence images for categorizing patients with glomerulonephritis. The model mainly comprises techniques like multimodal fusion, self-reliance coefficient extraction, and glomerular segmentation. This architecture primarily recognizes and segments the glomerulus, followed by glomerular methods trained to extract the features of every glomerulus. In [14], the authors developed a wide-ranging DL method. The developed architectures include three methods: survival prediction, clinical variable selection, and 3D image feature extractor. According to the 3D-CNN model, the feature extractor method forecasts the ISUP evaluation of RCC related to death rates in CT images. Clinical variables have been analytically chosen to employ the random forest (RF) and Spearman score significance score as standards. Chanchal et al. [15] examined a strong and computationally effective fully automatic RCC Grading Network (RCCGNet) method. This method comprises a shared channel residual (SCR) block that permits the model to learn feature maps related to various input forms in 2 parallel manners. The SCR block transfers the data between 2 diverse layers and functions the exchanged information individually by offering useful complements to one another.

In [16], a classification technique was developed by combining the features in a DL method with the removed texture features. Five texture features from 3 texture feature types have been implemented to match Alex-Net for the wide-ranging analysis of the combination of the texture and deep features. The texture features could be obtained from (i) statistic feature types like local binary pattern (LBP), grey-level cooccurrence matrix (GLCM), and histogram of gradient (HOG); (ii) transform based texture feature type such as Gabor filters; and (iii) system-based texture feature type: Markov random field (MRF). Mukashyaka et al. [17] projected an unsupervised and rapid SAMPLER technique. Image-level views of SAMPLER have been produced by encoding the collective distribution process of multiscale tile-level features. Besides, non-small cell lung carcinoma (NSCLC), slide-level representations of breast carcinoma (BRCA), and RCC-WSIs of The Cancer Genome Atlas (TCGA) have been implemented. Additionally, BRCA and NSCLC methods could be externally confirmed under frozen WSIs. In [18], a hybrid multi-instance learning system was developed, dependent upon the Transformer and Graph Attention Network (GAN) termed TGMIL. A feature pyramid called MMFP was primarily designed with several lower magnifications of WSI. Secondarily, TGMIL combines the abilities of TGMIL. Employing the GAN method, a simple and effective method utilizing the mean pooling and max pooling, produces the graph adjacency matrix. In conclusion, the outcomes of the two methods of TGMIL could be integrated. Kumar et al. [19] present an advanced approach for kidney disease detection using RF and gradient boosting (GB) approaches.

Uddin et al. [20] propose an ML method utilizing algorithms like support vector machine (SVM), Decision Tree (DT), RF, and k-nearest neighbour (KNN). Kadhim and Mohammed [21] develop ML models, SVM and MLP, for precise kidney cancer classification using CT images. It also utilizes GLCM and Gabor filters for feature extraction and data preprocessing to improve model reliability and prevent overfitting. Praveen et al. [22] propose an ML-based Neuro-Fuzzy model for early CKD prediction, utilizing image processing to detect fibrosis in kidney tissues. Singh and Gulati [23] aim to predict chronic diseases utilizing ML, specifically CNNs for feature extraction and K-nearest neighbour for classification. Data preprocessing, noise removal, and training/testing splits are employed to improve model accuracy. Zheng et al. [24] develop and evaluate four ML approaches, namely logistic regression (LR), RF, neural networks, and XGBoost, for predicting CKD progression, using AUC-ROC to assess model performance. Jayashree and Anitha [25] introduce a novel ML methodology incorporating quantum mutual correlation and radial basis extreme learning for chronic kidney disease (CKD) diagnosis. It extracts key features, utilizes quantum mutual information for selection, and uses radial basis extreme learning for classification, addressing data imbalance and computational challenges. Liu et al. [26] explore strategies for incorporating multi-omics data with ML to improve kidney disease prediction, understand molecular mechanisms, and apply image recognition in renal pathology. Rojas et al. [27] develop an interpretable ML techniques by utilizing XGBoost to predict glomerular filtration rate (GFR), integrating SHapley Additive exPlanation (SHAP) values for model interpretability and validation. Ghosh and Khandoker [28] develop a predictive model for CKD using ML methods, leveraging clinical data. For model interpretability, five ML techniques were utilized, with SHAP and Local Interpretable Model-agnostic Explanations (LIME).

Despite the improvements in ML for kidney disease prediction, various limitations and gaps remain. Many existing methods face difficulty with data imbalance, making it difficult to precisely predict outcomes in underrepresented groups. Furthermore, some models lack interpretability, which is significant for clinical adoption. There is also a requirement for more robust techniques to handle the high dimensionality of multi-omics and imaging data. Many approaches depend on a single input data type, whereas integrating several data sources such as clinical, demographic, and genomic information could improve model performance. Moreover, most studies focus on small datasets, limiting the generalizability and external validation of the models. Finally, there is restricted exploration of real-time prediction and deployment of these models in clinical settings.

## 3. The proposed method

**Fig 1:**
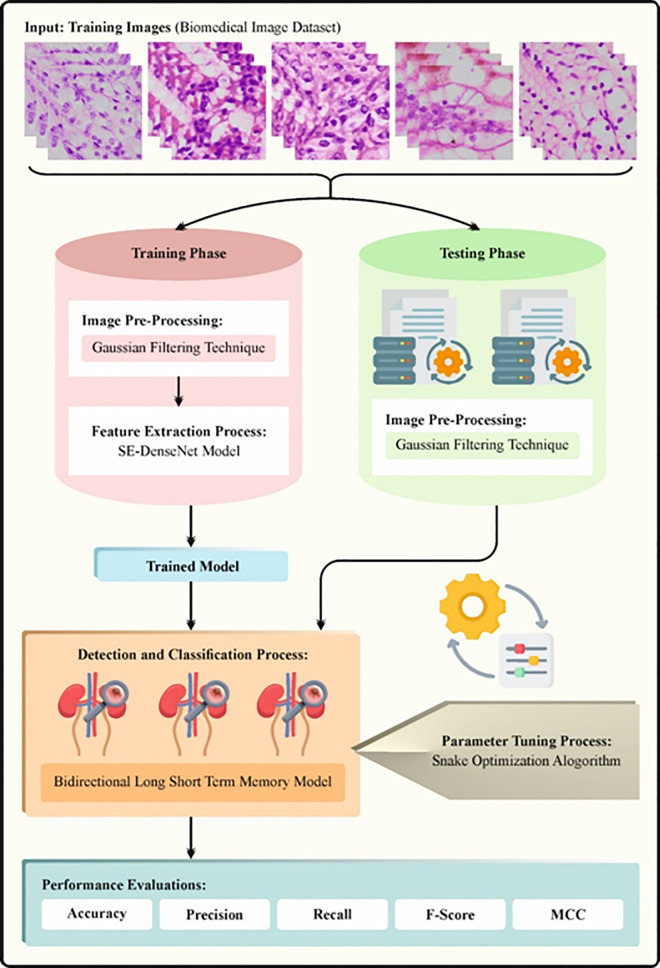
Workflow of KCDC-SODLPI technique.

This article develops the KCDC-SODLPI method, which analyzes pathological images to determine the presence of kidney cancer. The multifaceted process of the KCDC-SODLPI technique contains different processes, such as image preprocessing, SE-DenseNet-based feature extractor, SO-based parameter tuning, and BiLSTM-based classification. Fig 1 represents the workflow of the KCDC-SODLPI model.

### 3.1. GF-based image preprocessing

**Fig 2:**
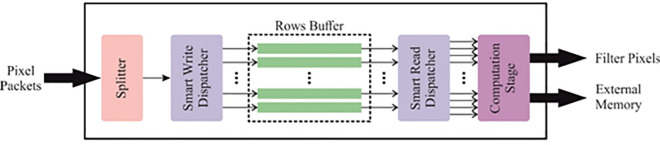
GF architecture.

Initially, the KCDC-SODLPI technique begins with a GF-based image preprocessing approach to eliminate the noise content [29]. This model is chosen for its capability to effectively mitigate noise while preserving significant image details, which is crucial for accurate kidney cancer detection in HIs. Unlike other methods that blur or distort critical features, GF smooths the image by averaging pixel values in a local neighbourhood, ensuring that the fine details related to cancerous patterns are retained. This preprocessing step improves the quality of input images, resulting in more reliable feature extraction in subsequent stages. Additionally, GF-based filtering is computationally efficient, making it ideal for massive datasets typical in medical imaging. Its simplicity and efficiency in removing high-frequency noise make it a preferred choice for cancer detection. Fig 2 demonstrates the structure of the GF model.

GF is a predominant image preprocessing method that can increase the quality of digital photos by mitigating noise and improving crucial features. This system exploits a GF kernel, a bell-shaped precise function with the input image. The convolution method employs a weighted average to the pixel values, with the weights determined by the Gaussian distribution. Higher-frequency noise will be effectively smoothed out, and the overall image should be more uniform and visually attractive. The primary significant benefit of GF is its capability to preserve critical structural details while lessening adverse artefacts. The smoothing impact accomplished through convolution is mainly valuable in decreasing the effect of random differences in pixel intensities like Gaussian noise or salt-and-pepper noise. This is particularly advantageous in these conditions where the raw image data will be degraded in transmission or acquisition.

### 3.2. SE-densenet feature extractor

**Fig 3:**
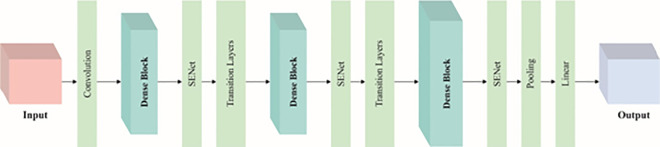
Steps involved in the SE-DenseNet technique.

The KCDC-SODLPI technique utilizes the SE-DenseNet model to extract intricate patterns of the input images [30]. This model was chosen for its capability to capture intrinsic patterns in HIs due to its dense connectivity and attention mechanism. DenseNet ensures effective feature propagation by connecting each layer to every other layer, enhancing gradient flow and mitigating the risk of vanishing gradients, which is critical when dealing with deep networks. The addition of the SE block improves this by adaptively recalibrating channel-wise feature responses, allowing the model to concentrate on more informative features. This makes SE-DenseNet appropriate for detecting subtle details in kidney cancer images, giving superior accuracy to conventional CNNs or even simpler DenseNet models. Integrating these advanced features results in enhanced performance in terms of accuracy and efficiency, making it an ideal choice for medical image analysis. Fig 3 illustrates the steps involved in the SE-DenseNet model.

DenseNet introduces the dense connectivity concept that can enhance the performance of a network without computation and increase parameters compared to classical network structures. There may be bottleneck data during information transmission in classical network structure because the neuron can only receive information from the feature map of the previous layer. In DenseNet, all the layers receive a feature map from each prior layer; hence, the data from the historical layer will be used better. Furthermore, dense connectivity can improve network training stability and generalization ability and help prevent gradient vanishing. Classical networks like ResNet use the dotted and solid residual connection to add the convolutional structure’s input and output, which is later fed into the following layer. The mapping equation is expressed as:


$$x_{l}=H_{l}\left(x_{l-1}\right)+x_{l-1}.$$ (1)

The output of ${the\;l}^{th}$ layer, as $x_{l}$, is attained by concatenating the output of each prior layer as $x_{l-1}$ and passing through a nonlinear function $H_{l}$. The dense connection enables each layer to be mutually connected and concatenates the output of all prior layers as the input to the following layer. The mapping formula is given by:


$$x_{l}=H_{l}\left(\left[x_{0},x_{1},\ldots ,\;x_{l-1}\right]\right).$$ (2)

In Eq. (2), $x_{l}$ denotes the output of $l^{th}$ layers, $\left[x_{0},\;x_{1}\ldots ,\;x_{l-1}\right]$ shows the output concatenation of the (l−1text−th layer and the result of each prior layer, and $H_{l}$ is a nonlinear conversion function.

The DenseNet has 3 Transition Layers and 4 DenseBlocks and is divided into various versions based on the number of pooling and convolutional layers, like DenseNetl69, DenseNet201, DenseNetl21, and so on. This study applies the small DenseNetl21 with fewer parameters and layers. The Transition Layer $T_{i}$ has pooling and convolutional layers connecting 2 Dense Blocks. The pooling layer compresses the model, and the convolutional layer decreases the sets of features. The Dense Block has 4 Dense Layers. First, the input is normalized using BN and passes over the activation function (ReLU), followed by the lxl convolutional layer to reduce computation cost. Later, it passes over another ReLU and BN and is given as output via 3×3 convolutional layers.

In the domain of CV, the Attention mechanism must be gaining popularity. The concept behind the attention mechanism is to devise a weight distribution for the original feature mapping and exploit it to the feature mapping. The SENet allocates weight to different image positions from the channel domain perspective to attain higher feature data via the weight matrix.


$$\begin{array}{c} Z_{c}=F_{sq}\left(u_{c}\right)=\frac{1}{H\times W}\sum_{i=1}^{H}\sum_{i=1}^{W}u_{c}(i,\;j) \end{array}$$ (3)

From Eq. (3), $u_{c}$ signifies the feature map of $C^{th}$ channels in the input feature; W and H describe the width and height correspondingly; $Z_{c}$ indicates the feature mapping value at the coordinate location. Once the SENet is embedded with the specific layer, the feature map received is subjected first to $F_{sq}$ function, where $F_{sq}$ refers to the global average pooling used to reduce the size of the feature map into lxl whilst C remains unchanged. Next, $\begin{array}{c} the\;Fex(,W) \end{array}$ function is implemented, which has sigmoid functions, activation functions, and FC layers that normalize the specific value of C. Lastly, after the SENet operation, F-scale is used for performing matrix multiplication between the original feature map and C, and the size is unchanged. After the convolution operation of the Dense Layer and the modified Dense Layer, SENet model is embedded into all the layers. After embedding SENet, the network integrates attention features, focuses more on important information during feature extraction, improves the model’s recognition rate, ignores irrelevant data such as background, and removes interference.

### 3.3. Hyperparameter tuning using SO model

**Fig 4:**
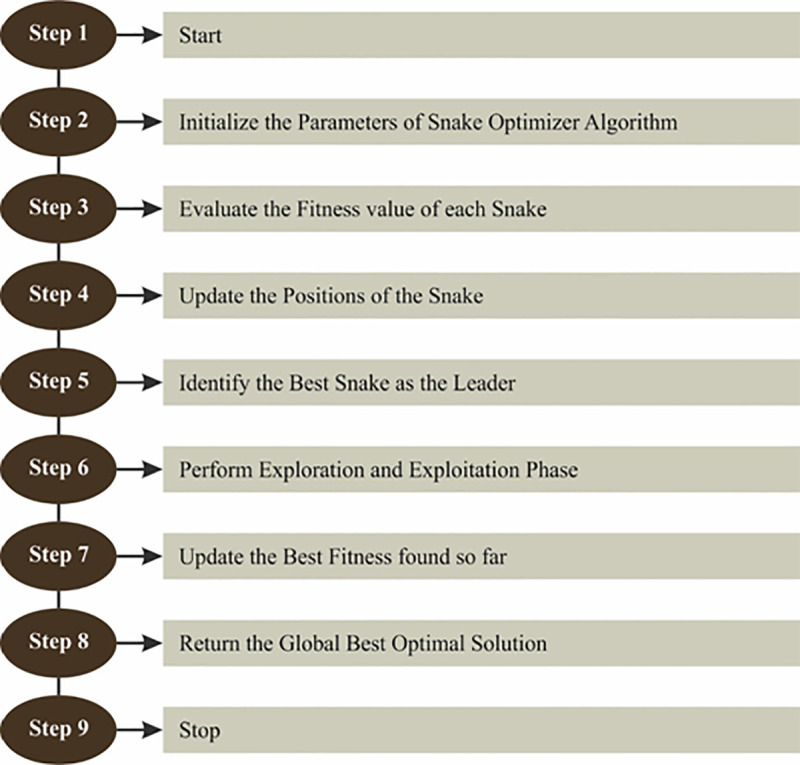
Structure of SO methodology.

The SO method performs the hyperparameter tuning of the SE-DenseNet model [31]. This model is chosen due to its effectual search mechanism and capability to navigate complex, high-dimensional spaces. SO replicates the movement of a snake, adjusting its position iteratively, which is highly efficacious for fine-tuning parameters in DL methods like SE-DenseNet. Unlike conventional optimization techniques such as grid or random search, SO dynamically adapts to the landscape of the problem, averting local minima and improving convergence toward optimal solutions. The exploration and exploitation balance of the model additionally ensures robust performance, enhancing accuracy and generalization. Additionally, the capability of the SO method to fine-tune hyperparameters with minimal computational overhead makes it an ideal choice, ensuring faster and more accurate results compared to other optimization methods. This results in an efficient and effective model optimization process. Fig 4 illustrates the SO structure.

SO is a recent metaheuristic approach stimulated by the particular mating behaviour of snakes. Temperature and food availability are substantial factors in snake behaviour, affecting mating and survival. Adequate food promotes reproductive health, and when food is abundant, snakes consider mating; however, if food is scarce, they focus on searching for it. Temperature also affects mating, with cooler environments encouraging female snakes to mate. This model has two phases: exploration and exploitation. During exploration, snakes search for food in response to environmental factors such as heat or food scarcity, while exploitation focuses on localized searches for better solutions. Mating occurs when there is sufficient food and cool temperatures, with males competing for females in combat mode, and females selecting the best mates. Successful mating leads to egg-laying and the hatching of new snakes.

The SO model initializes a population randomly within defined boundaries, segmenting it into two groups: males and females. Each individual’s position is updated based on fitness and a random number generator. The algorithm adapts the search strategy depending on temperature and food availability.

The temperature (Temp) decreases with the iteration count, influencing the exploration (when food is scarce, Q<0.25) and exploitation (when food is abundant, Q>0.25) strategies. When food is scarce, the positions of individuals are updated utilizing a randomized exploration equation, where males and females update their positions based on foraging strength.

For males:


$$X_{i,m}(t)=X_{rand,m}(t+1)\pm c2\times A_{m}\times \left(\left(X_{\max}-X_{\min}\right)\times rand+X_{\min}\right)$$ (4)

$$A_{m}=exp\left(\frac{-f_{rand,m}}{f_{i,m}}\right)$$ (5)

In Eq. (4), the location of the $i^{th}$ male individual is $X_{i,m}$, the area of randomized male individuals is $X_{rand,m}$, and rand is a random number within [0,1]. In Eq. (5), $A_{m}$denotes the foraging strength of male individuals, the fitness of $X_{rand,m}$ is $f_{rand,m},\;f_{i,m}$ denotes the fitness of $i^{th}\;male\;$individuals in the population, and a constant c2 is set as 0.05. When the environment is hot (Temp>0.6), individuals move towards the food, with position updates based on food location.

For females:


$$X_{if}(t)=X_{rand,f}(t)\pm c2\times A_{f}\times \left(\left(X_{\max}-X_{\min}\right)\times rand+X_{\min}\right)$$ (6)

$$A_{f}=exp\left(\frac{-f_{rand,f}}{f_{i,f}}\right)$$ (7)

In Eq. (6), the location of ${the\;i}^{th}$ female is $X_{i,f}$, the random area of the female is $X_{rondf}$, rand indicates the random integer within [0,1]. In Eq. (7), the foraging power of female individuals is $A_{f}$, $f_{i,f}$ denotes the fitness of $i^{th}$ female individuals in the population, $f_{rand,f}$ refers to the fitness of $X_{rand,\;f},\;$ and a constant c2 is set to 0.05. Snakes are cryogenically reproduced creatures. The temperature ranges between [0,1]. If Q>0.25, then the food is sufficient, and the temperature condition is considered.

Position update towards food:


$$X_{i,j}(t+1)=X_{food}\pm c3\times Temp\times rand\times \left(X_{food}-X_{i,j}(t)\right)$$ (8)

In Eq. (8), the individual position is represented as $X_{ij}$, $X_{food}$ is the fittest individual location, and a constant c3 is set as 2. In cooler conditions (Temp<0.6), the algorithm switches to a combat or mating pattern. For combat:

Combat position updates for males:


$$X_{i,m}(t+1)=X_{i,m}(t)+c3\times FM\times rand\times \left(Q\times X_{best,f}-X_{i,m}(t)\right)$$ (9)

Combat position updates for females:


$${\;X}_{i,f}(t+1)=X_{i,f}(t+1)+c3\times FF\times rand\times \left(Q\times X_{best,m}-X_{i,f}(t+1)\right)$$ (10)

In Eq. (9), the location of ${the\;i}^{th}$ male is denoted as $X_{i,m}$, $X_{best,f}$, indicating the location of the fittest individuals among females, and the fighting strength of males is expressed as FM. In Eq. (10), the area of $i^{th}$ female is $X_{if}$, $X_{best,m}$ signifies the location of fittest individuals amongst males, and FF means the fighting strength of females. FM and FF are calculated using the fitness values of the best individuals in the male and female populations:

FM and FF:


$$FM=\exp\left(\frac{-f_{best,f}}{f_{i}}\right)$$ (11)

$$FF=\exp\left(\frac{-f_{best,m}}{f_{i}}\right)$$ (12)

In Eqs. (11–12), the fitness values of individuals in the female and male groups are denoted as $f_{best,f}$ and $f_{best}$, and m, and the individual fitness values are indicated as $f_{i}$. Finally, mating behaviour is incorporated, and individuals update positions based on mating capabilities $M_{m}$ and $M_{f}$:

Mating position updates:


$$X_{i_{i}m}(t+1)=X_{i,m}(t)+c3\times M_{m}\times rand\times \left(Q\times X_{if}(t)-X_{i,\;m}(t)\right)$$ (13)

$$X_{i,f}(t+1)=X_{i,f}(t)+c3\times M_{f}\times rand\times \left(Q\times X_{i,m}(t)-X_{if}(t)\right)$$ (14)

In Eqs. (13–14), $X_{i,\;m}$, and${\;X}_{if}$ are the positions of $i^{th}$ males and females in the population, and $M_{m}{\;and\;M}_{f}$ are the mating capabilities of males and females, respectively.

In summary, the SO algorithm optimizes individuals’ positions based on environmental conditions, foraging strength, and fitness, balancing exploration and exploitation to find the optimal solution.

Fitness selection is a significant parameter that manipulates the effectiveness of the SO method. The hyperparameter selection method comprises the encoding system for evaluating the efficiency of the candidate outcomes. During this study, the SO method deliberates accuracy as the critical measure to calculate the fitness function (FF) represented below.


Fitness = max (P) (15)

$$P=\frac{TP}{TP+FP}\;$$ (16)

In mathematical expression, FP means the false positive, and TP signifies the true positive values.

### 3.4. BiLSTM-based classification process

**Fig 5:**
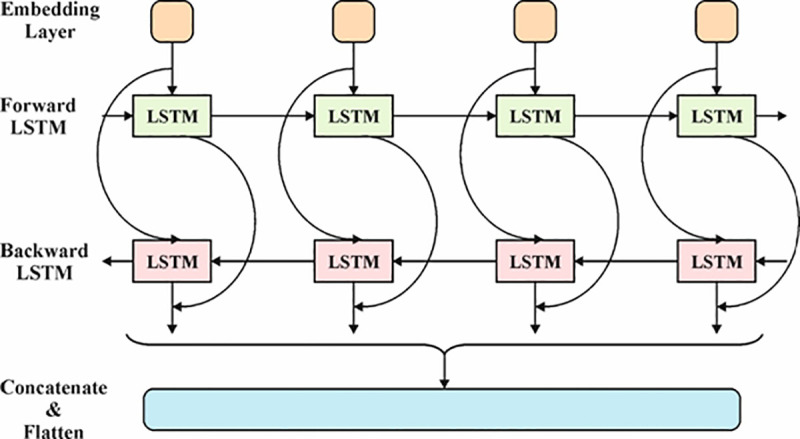
Architecture of BiLSTM.

In conclusion, the BiLSTM model detects and classifies kidney cancer [32]. This model is chosen because it can capture both past and future dependencies in sequential data. Unlike conventional LSTM models that only capture data from the past, BiLSTM processes data in both forward and backward directions, improving its understanding of temporal patterns and accuracy. This bidirectional approach benefits complex tasks like kidney cancer detection, where the relationship between diverse image features can be temporal and spatial. Moreover, the capacity of the BiLSTM model to effectively handle long-range dependencies assists in recognizing subtle discrepancies in HIs, resulting in more accurate classification. Compared to simpler models, BiLSTM is better suited for tasks requiring context-aware decision-making, thus giving superior performance in this application. Fig 5 illustrates the substructure of BiLSTM.

The Bi-LSTM block is the second module in the technique, comprising of a BiLSTM network, dense, and dropout layers. LSTM, a type of RNN, addresses the long-term dependency issue found in classical RNNs by using memory cells to store relevant data. Each LSTM unit includes three gates and a memory cell, which assists in controlling data flow within the hidden layer (HL). The gates determine what data should be forgotten or retained, allowing the model to efficiently process time-series data and capture long-term dependencies. Acquiring long‐term dependencies by LSTM and resolving the before-stated problem is highly crucial. The input, output, and forget are three types of gates. Then, their characteristics are briefly defined. The sigmoid function is used for data in the current input $\left(X_{t}\right)$ and the preceding HL (h−1) in the forget gate. This task is signified by $f_{t}$, which yields a value between zero and one, specifying the ratio of data that must be retained.

The input gate takes the data in the existing input and preceding HL and then permits them over 2^nd^ sigmoid function by altering this data into a value between zero and one. Also, similar data permits a function of tanh that aids in controlling the system, returning a value between −1 and 1. Next, the sigmoid output $\left(i_{t}\right)$ increased by the tanh output $\left({\tilde{C}}_{t}\right)$ to choose which data is significant to save. Now, sufficient data is utilized to calculate the cell state. At initial, the preceding cell state $\left(C_{t-1}\right)$ has been multiplied by the output of the forget gate. Then, the input gate output was included, upgrading the cell state with novel values that were measured related by the system. The outcome of these dual processes is the novel cell state $\left(C_{t}\right)$.

Lastly, the output gate selects the value of subsequent HL. At initial, the data from the existing input and preceding HL permits over a 3^rd^ sigmoid function. Next, the novel cell state licenses over a tanh function. Both outputs have been multiplied point-wise. The yield of the output gate $\left(0_{t}\right)$ is the novel HL $\left(h_{t}\right)$. So, the new cell state and HL will be taken to the next time step. Officially, the processes that occur in an LSTM are given below:


$$\begin{array}{c} f_{t}=\sigma \left(W_{f}\cdot \left[h_{t-1},\;x_{t}\right]+b_{f}\right)\\ i_{t}=\sigma \left(W_{i}\cdot \left[h_{t-1},\;x_{t}\right]+b_{i}\right)\\ \begin{array}{c} {\tilde{C}}_{t}=tanh\;\left(W_{c}\cdot \left[h_{t-1},\;x_{t}\right]+b_{C}\right)\\ C_{t}=f_{t}\cdot C_{t-1}+i_{t}\cdot {\tilde{C}}_{t}\\ \begin{array}{c} o_{t}=\sigma \left(W_{o}\left[h_{t-1},\;x_{t}\right]+b_{o}\right)\\ h_{t}=o_{t}\cdot \tanh\left(C_{t}\right) \end{array} \end{array} \end{array}$$ (17)

Here, $x_{t}$ means the input data in time t; σ and tanh signify a sigmoid and hyperbolic tangent function, respectively; $h_{t}$ embodies the HL in time t, $W_{x}$, and $b_{\chi }$ signifies a weight and bias, respectively. After clarifying the performance of an LSTM model, the BiLSTM network functions must be defined. It can generally be the development of LSTM dependent upon bidirectional RNN. A BiLSTM model has a forward and backward LSTM, which uses input values ranging from t−k to t and t to t−k, respectively. The backward $\left(\overset{\leftarrow }{h}\right)$ and forward $(\vec{h}\backslash )$network outputs have been calculated using the device defined beforehand for one LSTM. The BiLSTM layer yields a vector $\left(y_{t}\right)$, which is attained by using the mentioned expression:


$$Y_{t}=\sigma \left({\vec{h}}_{t},\;{\overset{\leftarrow }{h}}_{t}\right)$$ (18)

Where a sigmoid function unites the single LSTM network output, it is noticeable that BiLSTM uses a dropout method to control overfitting. The rate of dropout has a hyperparameter to study in the description of this method. Lastly, a fully connected (FC) layer that predicts the outputs is loaded.

## 4. Performance validation

**Fig 6:**
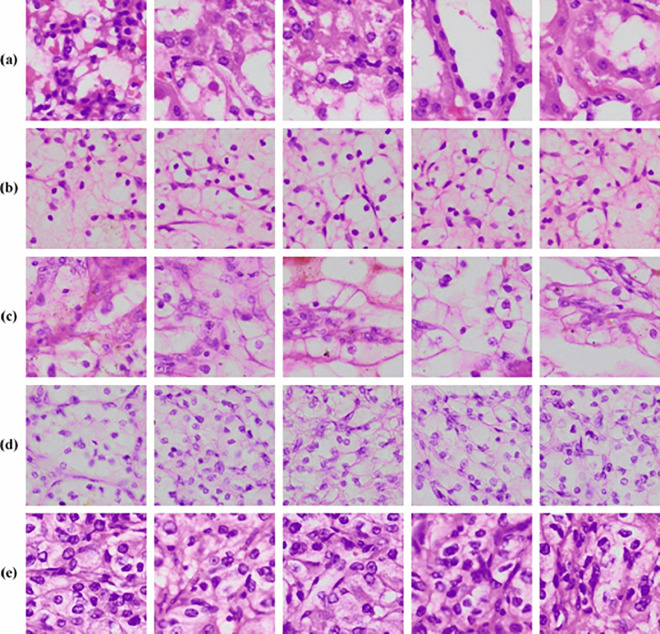
Sample Images a) Grade-0 b) Grade-1 c) Grade-2 d) Grade-3 e) Grade-4.

The validation evaluation of the KCDC-SODLPI method is examined by employing a biomedical image dataset [15,33]. The dataset encompasses 3000 instances with five classes, as defined in Table 1. Grade-0 (Cells are well organized and are expected in number), Grade-1 (Nucleoli are not noticeable even 400 × magnification Nucleoli are realized as eosinophilic at 400 × magnification and not more prominent at 100 × magnification), Grade-2 (moderately unequal contour compared to normal nuclei), Grade-3(Clearly visible cancers represents graded as grade-3), Grade-4(Rhabdoid or sarcomatoid difference). Fig 6 illustrates the instances images.

**Table 1:** Details on database.

Type	Labels	No. of Images
Non-Cancerous	Grade-0	600
Cancerous	Grade-1	600
	Grade-2	600
	Grade-3	600
	Grade-4	600
**Total No. of Images**		**3000**

**Fig 7:**
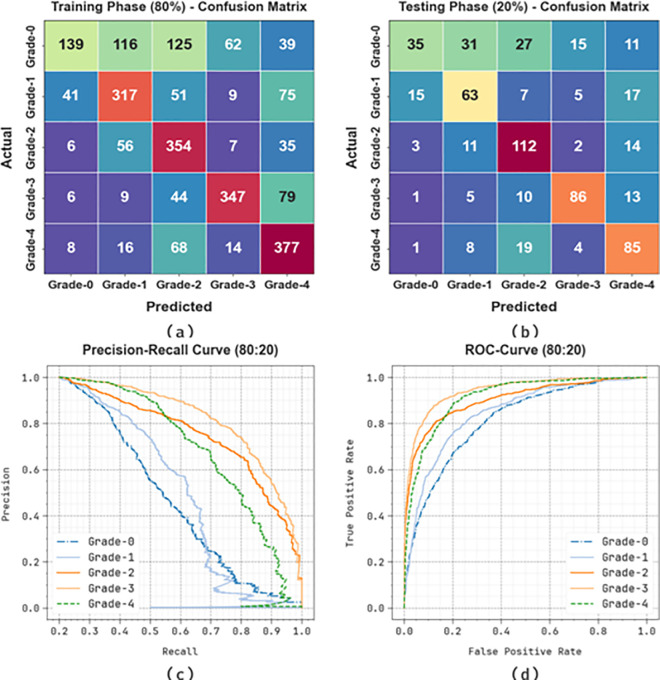
80%TRAS and 20%TESS (a-b) Confusion matrices and (c-d) PR and ROC curves.

Fig 7 displays the classifier outcomes of the KCDC-SODLPI method at 80%TRAS and 20%TESS. Fig 7a-7b showcases the confusion matrices made by the KCDC-SODLPI method. This figure indicates that the KCDC-SODLPI method is exactly recognized and categorized into five classes. Moreover, Fig 7c showcases the PR result of the KCDC-SODLPI technique. This figure shows that the KCDC-SODLPI technique gains higher PR effectiveness with five classes. In conclusion, Fig 7d exemplifies the ROC result of the KCDC-SODLPI technique. This figure showed that the KCDC-SODLPI technique provides effective outcomes with increased ROC values in 5 classes.

**Fig 8:**
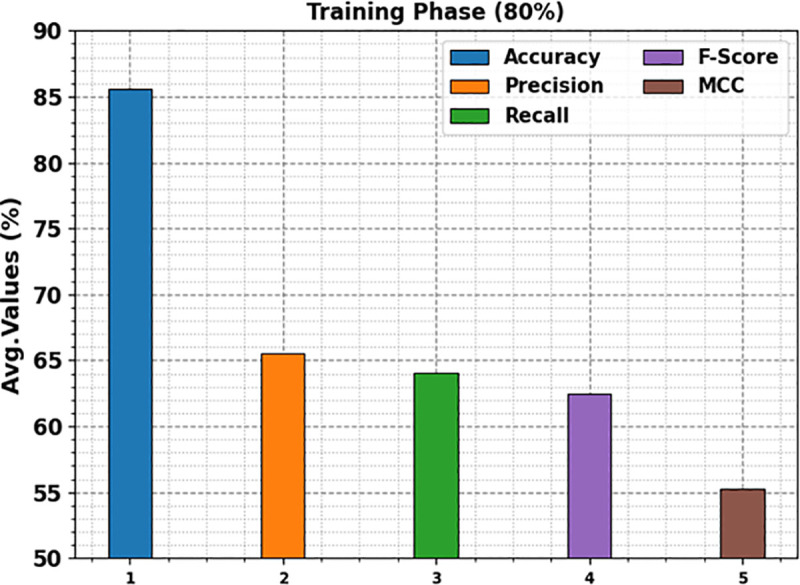
Average of KCDC-SODLPI technique under 80% of TRAS.

In Table 2, the overall kidney cancer detection results of the KCDC-SODLPI model are under 80%TRAS and 20%TESS. Fig 8 represents the average outcomes of the KCDC-SODLPI technique with 80% TRAS. The figure illustrates that the KCDC-SODLPI technique gains productive performance when recognizing five classes. It is also noticed that the KCDC-SODLPI technique accomplishes an average $accu_{y}$ of 85.57%, $prec_{n}$ of 65.53%, $reca_{l}$ of 64.02%, $F_{score}$ of 62.51%, and MCC of 55.25%, respectively.

**Table 2:** Kidney cancer detection outcome of KCDC-SODLPI method at 80%TRAS and 20%TESS.

Classes	$Accu_{y}$	$Prec_{n}$	$Reca_{l}$	$F_{Score}$	MCC
**TRAS (80%)**					
Grade-0	83.21	69.50	28.90	40.82	37.25
Grade-1	84.46	61.67	64.30	62.96	53.15
Grade-2	83.67	55.14	77.29	64.36	55.45
Grade-3	90.42	79.04	71.55	75.11	69.32
Grade-4	86.08	62.31	78.05	69.30	61.09
**Average**	**85.57**	**65.53**	**64.02**	**62.51**	**55.25**
**TESS (20%)**					
Grade-0	82.67	63.64	29.41	40.23	34.90
Grade-1	83.50	53.39	58.88	56.00	45.96
Grade-2	84.50	64.00	78.87	70.66	60.89
Grade-3	90.83	76.79	74.78	75.77	70.13
Grade-4	85.50	60.71	72.65	66.15	57.39
**Average**	**85.40**	**63.71**	**62.92**	**61.76**	**53.85**

**Fig 9:**
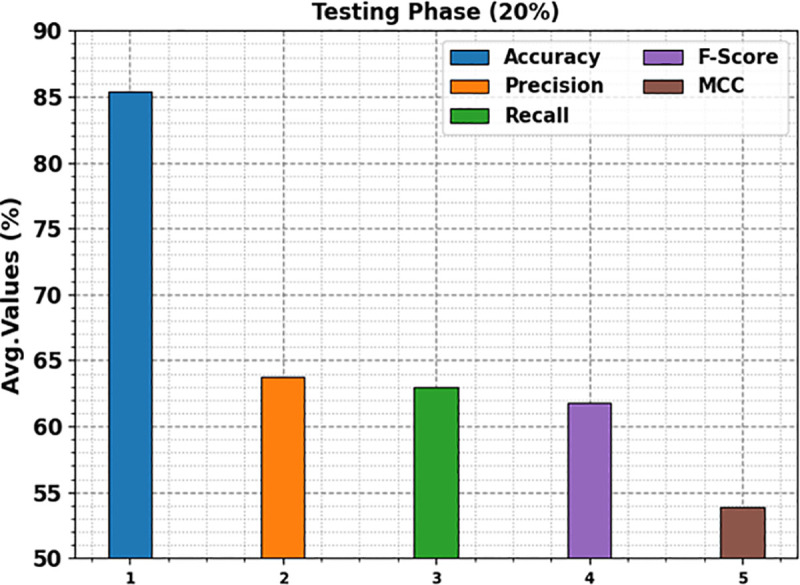
Average of KCDC-SODLPI model on 20% of TESS.

Fig 9 examines the average outcomes of the KCDC-SODLPI method with 20% TESS. The figure demonstrates that the KCDC-SODLPI method performs proficiently in recognizing five classes. The KCDC-SODLPI technique attains an average $accu_{y}$ of 85.40%, $prec_{n}$ of 63.71%, $reca_{l}$ of 62.92%, $F_{score}$ of 61.76%, and MCC of 53.85%, correspondingly.

**Fig 10:**
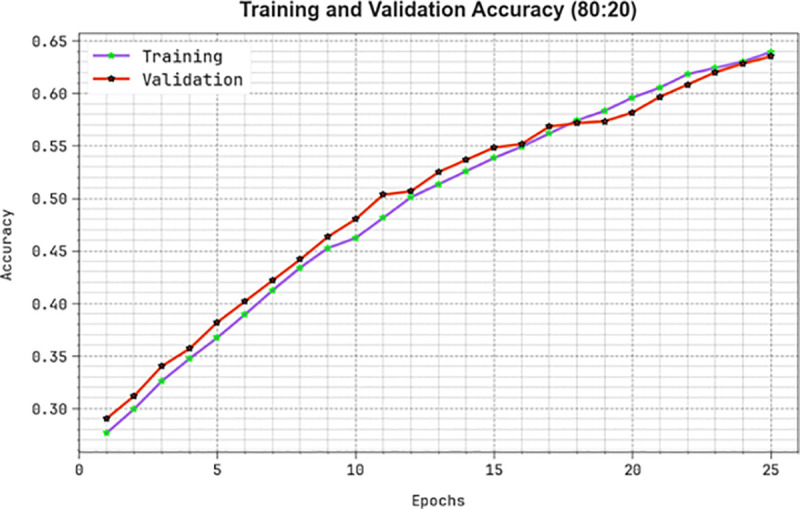
*Accu^y^* curve of KCDC-SODLPI method on 80%TRAS and 20%TESS.

The efficiency of the KCDC-SODLPI model at 80%TRAS and 20%TESS is graphically demonstrated in Fig 10 in the form of training accuracy (TRAA) and validation accuracy (VALA) curves. This figure displays a valuable analysis of the behaviour of the KCDC-SODLPI technique over varying epoch counts, representing its learning method and generalization capabilities. The figure assumes a constant improvement in the TRAA and VALA, with progress in epochs. It confirms the adaptive aspect of the KCDC-SODLPI technique in the pattern recognition process under TRA and TES data. The increased trends in VALA outline the capability of the KCDC-SODLPI technique to adapt to the TRA data and provide precise classification of unnoticed data, pointing out robust generalization abilities.

**Fig 11:**
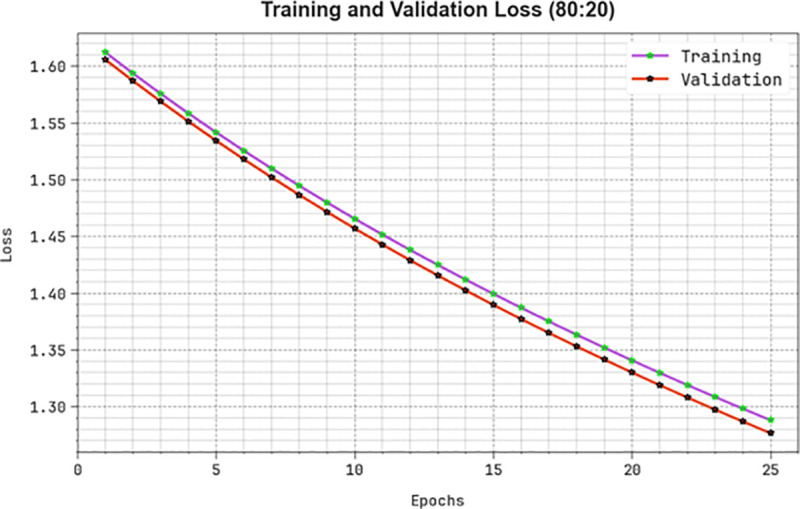
Loss curve of KCDC-SODLPI model under 80%TRAS and 20%TESS.

Fig 11 illustrates an extensive view of the training loss (TRLA) and validation loss (VALL) results of the KCDC-SODLPI technique on 80%TRAS and 20%TESS over distinct epochs. The progressive minimization in TRLA highlights the KCDC-SODLPI method, enhancing the weights and diminishing the classification error on the TRA and TES data. The figure specifies a perfect understanding of the KCDC-SODLPI method related to the TRA data, highlighting its proficiency in capturing patterns. The KCDC-SODLPI method significantly improves its parameters in decreasing the differences between the prediction and real TRA classes.

**Fig 12:**
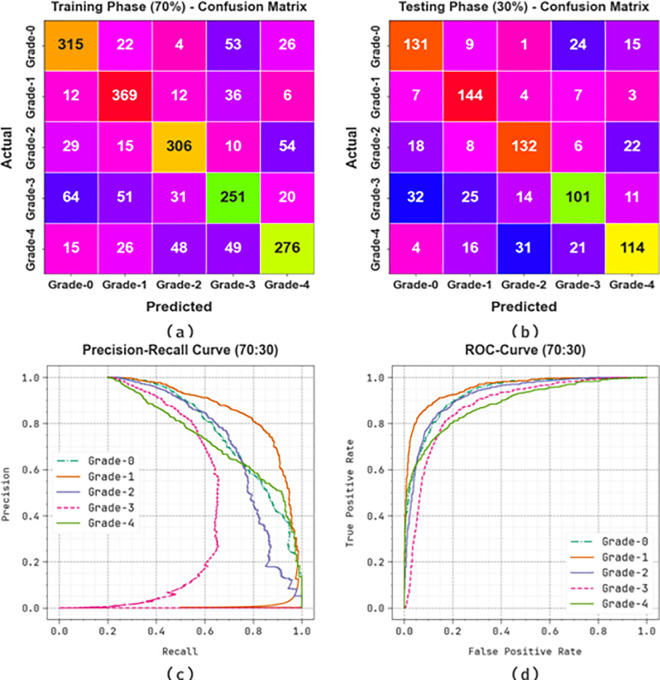
70%TRAS and 30%TESS (a-b) Confusion matrices and (c-d) PR and ROC curves.

Fig 12 examines the classifier analysis of the KCDC-SODLPI method with 70%TRAS and 30%TESS. Fig 12a-12b represents the confusion matrices offered by the KCDC-SODLPI method. The figure signified that the KCDC-SODLPI method can correctly identify and categorize with five classes. Meanwhile, Fig 12c demonstrates the PR result of the KCDC-SODLPI method. This figure specifies that the KCDC-SODLPI method has gained greater PR effectiveness in five classes. Also, Fig 12d showcases the ROC result of the KCDC-SODLPI method. This figure shows that the KCDC-SODLPI technique offers efficient outcomes with increased ROC values on five classes.

**Fig 13:**
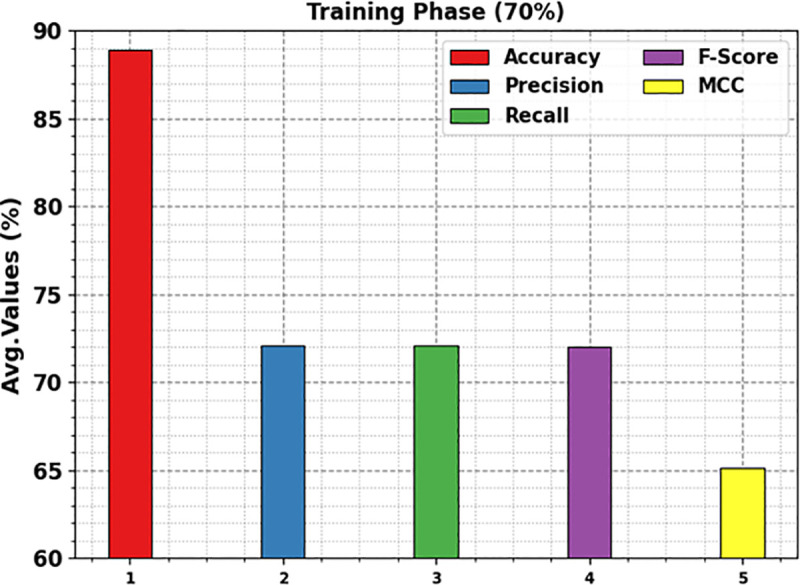
Average of KCDC-SODLPI model at 70% of TRAS.

A detailed overall kidney cancer detection outcomes of the KCDC-SODLPI method at 70%TRAS and 30%TESS are described in Table 3. Fig 13 displays the average outcomes of the KCDC-SODLPI method with 70% TRAS. The figure exhibits that the KCDC-SODLPI technique provides effectual performance under the recognition of five classes. This is also remarked that the KCDC-SODLPI technique realizes an average $accu_{y}$ of 88.90%, $prec_{n}$ of 72.06%, $reca_{l}$ of 72.12%, $F_{score}$ of 72.01%, and MCC of 65.14%, respectively.

**Table 3:** Kidney cancer detection outcome of KCDC-SODLPI technique under 70%TRAS and 30%TESS.

Classes	$Accu_{y}$	$Prec_{n}$	$Reca_{l}$	$F_{Score}$	MCC
**TRAS (70%)**					
Grade-0	89.29	72.41	75.00	73.68	66.98
Grade-1	91.43	76.40	84.83	80.39	75.09
Grade-2	90.33	76.31	73.91	75.09	69.11
Grade-3	85.05	62.91	60.19	61.52	52.27
Grade-4	88.38	72.25	66.67	69.35	62.27
**Average**	**88.90**	**72.06**	**72.12**	**72.01**	**65.14**
**TESS (30%)**					
Grade-0	87.78	68.23	72.78	70.43	62.79
Grade-1	91.22	71.29	87.27	78.47	73.62
Grade-2	88.44	72.53	70.97	71.74	64.48
Grade-3	84.44	63.52	55.19	59.06	49.71
Grade-4	86.33	69.09	61.29	64.96	56.66
**Average**	**87.64**	**68.93**	**69.50**	**68.93**	**61.45**

**Fig 14:**
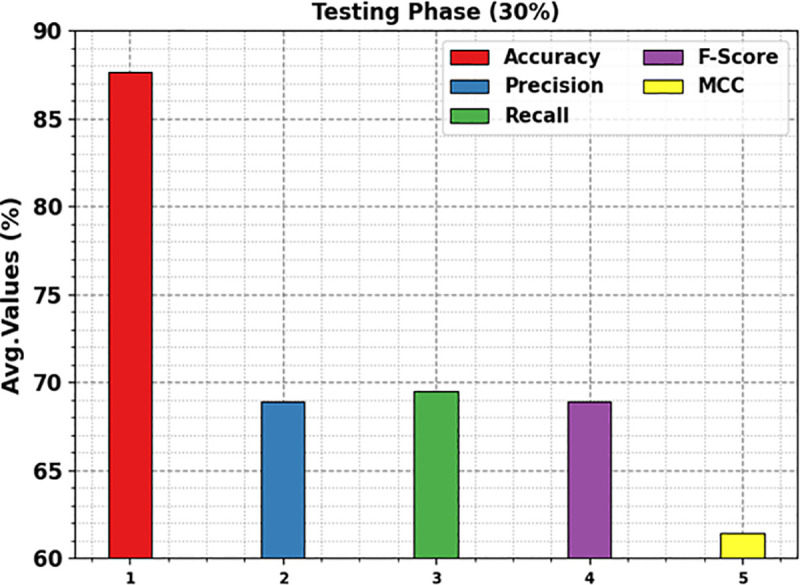
Average of KCDC-SODLPI technique under 30% of TESS.

Fig 14 shows the average results of the KCDC-SODLPI method with 30% TESS. The figure explains that the KCDC-SODLPI method performs excellently in recognizing five classes. This can also be perceived that the KCDC-SODLPI approach achieves an average $accu_{y}$ of 87.64%, $prec_{n}$ of 68.93%, $reca_{l}$ of 69.50%, $F_{score}$ of 68.93%, and MCC of 61.45%.

**Fig 15:**
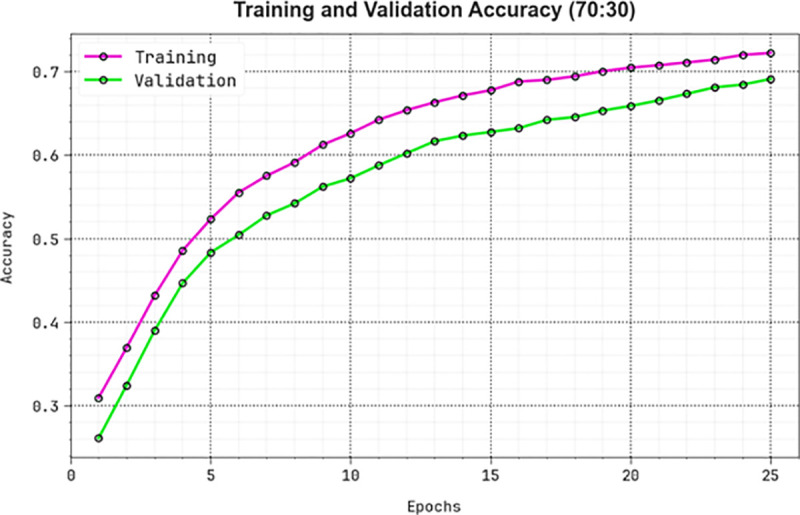
*Accu^y^* curve of KCDC-SODLPI technique on 70%TRAS and 30%TESS.

The efficiency of the KCDC-SODLPI method at 70%TRAS and 30%TESS is graphically reported in Fig 15 in the form of TRAA and VALA curves. The figure displays a functional interpretation of the behaviour of the KCDC-SODLPI technique over varying epoch counts, representing its learning process and generalization abilities. Notably, the figure steadily heightens the TRAA and VALA with progress in epochs. It ensures the adaptive aspect of the KCDC-SODLPI method in the pattern recognition process at the TRA and TES data. The higher trends in VALA outline the capability of the KCDC-SODLPI method to adapt to the TRA data and also surpass in offering precise classification on unnoticed data, exhibiting robust generalization abilities.

**Fig 16:**
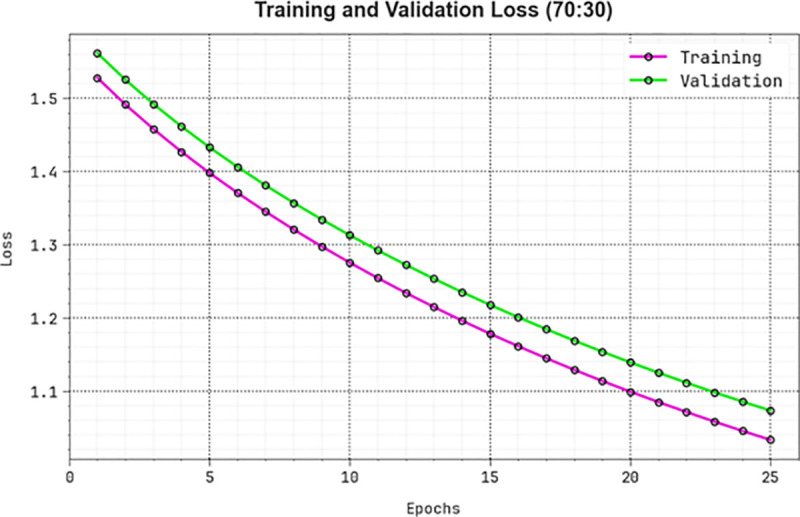
Loss curve of KCDC-SODLPI method at 70%TRAS and 30%TESS.

Fig 16 represents the TRLA and VALL results of the KCDC-SODLPI technique under 70%TRAS and 30%TESS over distinct epochs. The progressive reduction in TRLA highlights the KCDC-SODLPI technique, which improves weights and reduces classification errors with TRA and TES data. The figure specifies a better understanding of the KCDC-SODLPI method relevant to the TRA data, highlighting its proficiency in capturing patterns. The KCDC-SODLPI method continually improves its parameters, lessening the differences between the prediction and real TRA classes.

**Fig 17:**
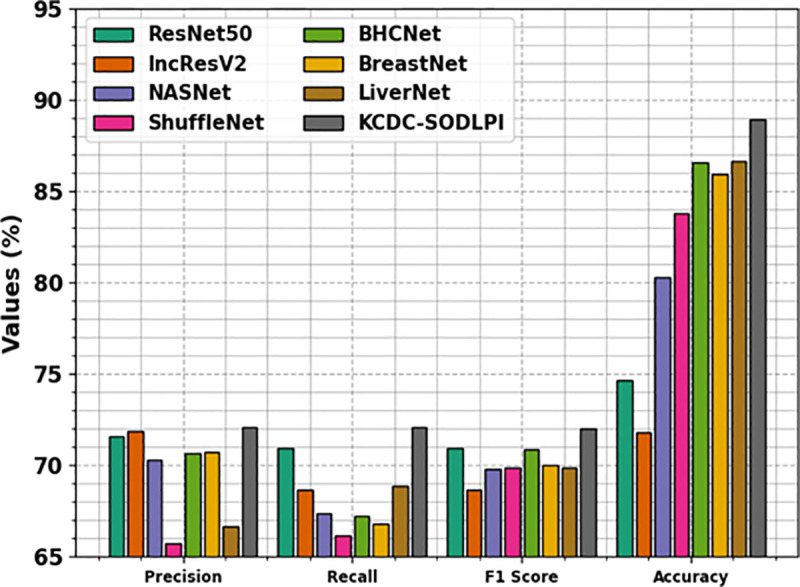
Comparative outcomes of the KCDC-SODLPI technique with recent approaches.

Table 4 and Fig 17 [15] provide a wide-ranging comparative result of the KCDC-SODLPI method with recent techniques. The results highlighted that the ShuffleNet and LiverNet models have shown the lowest performance over other models. The ResNet50, IncResV2, NASNet, BHCNet, and BreastNet models have shown considerable performance. However, the KCDC-SODLPI technique gains maximum performance over others with increased $prec_{n}\;$of 72.06%, $reca_{l}$ of 72.12%, $F1_{score}$ of 72.01%, and $accu_{y}$ of 88.90%.

**Table 4:** Comparative analysis of the KCDC-SODLPI technique with recent approaches [15].

Metrics	$Prec_{n}$	$Reca_{l}$	$F1_{Score}$	$Accu_{y}$
ResNet50	71.60	70.97	70.93	74.64
IncResV2	71.85	68.64	68.69	71.83
NASNet	70.30	67.37	69.79	80.28
ShuffleNet	65.74	66.13	69.87	83.80
BHCNet	70.65	67.20	70.86	86.61
BreastNet	70.76	66.81	70.05	85.91
LiverNet	66.69	68.84	69.90	86.62
KCDC-SODLPI	72.06	72.12	72.01	88.90

Therefore, the KCDC-SODLPI technique is used to automate the detection and classification of kidney cancer on medical images.

**Fig 18:**
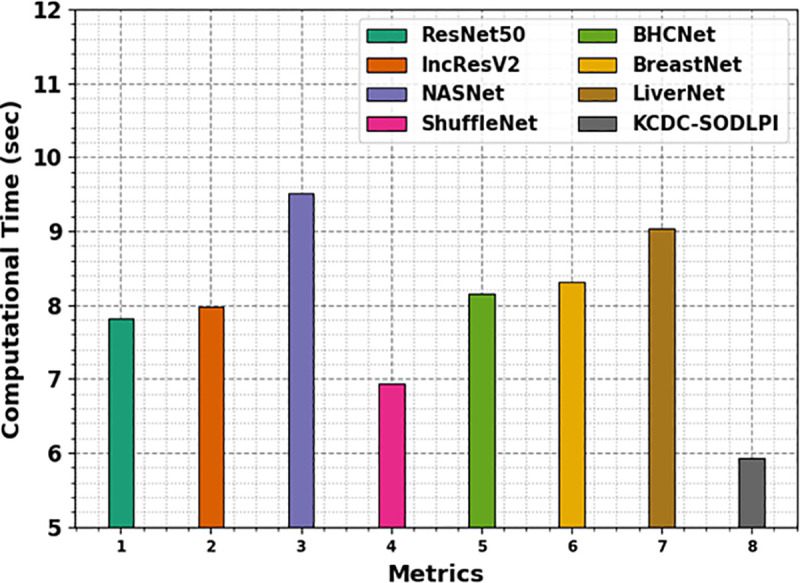
CT evaluation of the KCDC-SODLPI technique with existing models.

The performance of several models is evaluated in terms of computational time (CT), measured in seconds in Table 5 and Fig 18, with the results indicating the efficiency of each model. ResNet50, IncResV2, NASNet, and other models, such as BHCNet, BreastNet, and LiverNet, exhibit varying CTs, ranging from 6.93 to 9.52 seconds. Notably, the KCDC-SODLPI model outperforms all the other models with the lowest CT of 5.93 seconds, emphasizing its superior efficiency. This faster CT suggests that the KCDC-SODLPI model is effective in detecting and classifying kidney cancer and optimized for quicker processing, making it appropriate for real-time applications in clinical settings.

**Table 5:** CT evaluation of the KCDC-SODLPI technique with existing models.

Metrics	CT (sec)
ResNet50	7.81
IncResV2	7.98
NASNet	9.52
ShuffleNet	6.93
BHCNet	8.16
BreastNet	8.32
LiverNet	9.04
KCDC-SODLPI	5.93

## 5. Conclusion

In this article, the KCDC-SODLPI method is developed. The primary aim of the KCDC-SODLPI technique is to analyze the pathological images to determine the presence of kidney cancer. The KCDC-SODLPI technique employs a GF-based image preprocessing approach to eliminate the noise content in the multifaceted process. Besides, the KCDC-SODLPI method utilizes the SE-DenseNet model to extract intricate patterns from the input images. Moreover, the SO model is used to tune the hyperparameter of the SE-DenseNet method. Finally, the BiLSTM model is used to detect and classify kidney cancer. The performance of the KCDC-SODLPI technique is evaluated under the biomedical image dataset. The experimental validation of the KCDC-SODLPI method portrayed a superior accuracy value of 88.90% over existing models. The limitations of the KCDC-SODLPI method comprise various key challenges that affect its broader application. First, the dataset used for model training may not be diverse enough to represent the full spectrum of CKD patients, limiting the generalizability of the results. Furthermore, despite the high accuracy, the model’s performance may decrease when applied to real-world clinical settings due to variability in data quality and patient demographics. Moreover, the models could face difficulty handling missing or incomplete data, which is common in healthcare datasets. The lack of interpretability in some ML models also hinders clinical adoption. Future work should expand datasets to comprise a broader range of populations, enhance model robustness in real-world conditions, and incorporate explainable AI techniques to improve trust and usability in clinical decision-making. Furthermore, exploring real-time prediction systems and integrating longitudinal data could improve early CKD detection and management.
